# Expression of recombinant *Clostridium difficile *toxin A and B in *Bacillus megaterium*

**DOI:** 10.1186/1471-2180-8-192

**Published:** 2008-11-06

**Authors:** Guilin Yang, Boping Zhou, Jufang Wang, Xiangyun He, Xingmin Sun, Weijia Nie, Saul Tzipori, Hanping Feng

**Affiliations:** 1 Division of Infectious Diseases, Department of Biomedical Sciences, Tufts University Cummings School of Veterinary Medicine, North Grafton, Massachusetts 01536, USA; 2 Institute of Hepatology, Shenzhen East Lake Hospital, Shenzhen518020, PR China; 3 The Department of Biochemical Engineering, School of Bioscience and Biotechnology, South China University of Technology (SCUT), Guangzhou 510006, PR China

## Abstract

**Background:**

Major *Clostridium difficile *virulence factors are the exotoxins TcdA and TcdB. Due to the large size and poor stability of the proteins, the active recombinant TcdA and TcdB have been difficult to produce.

**Results:**

The toxin genes *tcdA *and *tcdB *were amplified by PCR using chromosomal DNA from a toxigenic strain as a template, and cloned into a shuttle vector pHis1522. The sequences of both *tcdA *and *tcdB *genes in the vector have been verified by DNA sequencing. The constructs were transformed into *B. megaterium *protoplasts and the protein expression was controlled under a xylose promoter. The recombinant toxins (rTcdA and rTcdB) were purified from bacterial crude extracts. Approximately 5 – 10 mg of highly purified recombinant toxins were obtained from one liter of bacterial culture. The resulting rTcdA and rTcdB had similar molecular masses to the native toxins, and their biological activities were found to be similar to their native counterparts after an extensive examination.

**Conclusion:**

We have generated the full length and active recombinant TcdA and TcdB in *Bacillus megaterium*.

## Background

*Clostridium difficile *is a Gram-positive, spore-forming anaerobic bacillus. It is the most common cause of nosocomial antibiotic-associated diarrhea and the etiologic agent of pseudomembranous colitis [1]. Antibiotic usage results in a reduction of commensal microflora in the gut, which permits *C. difficile *to proliferate more extensively, leading to the production of toxins [2]. *C. difficile *associated diarrhea (CDAD) include a range of symptoms varying from mild diarrhea to severe fulminate lethal disease [3]. Recent outbreaks of highly virulent *C. difficile *strains [4,5] have increased the urgency to devote greater resources towards the understanding of the molecular, genetic, and biochemical basis for the pathogenesis, with a view to use such information to develop novel preventative and treatment modalities.

Two exotoxins produced by toxigenic *C. difficile*, toxin A (TcdA) and toxin B (TcdB), are most extensively studied and thought to be major virulent factors of CDAD [6,7]. TcdA (308 kDa) and TcdB (269 kDa) belong to the large clostridial cytotoxin (LCT) family and share 49% amino acid identity [8]. The two toxins have a similar structure containing a putative receptor binding domain (RBD), a transmembrane domain (TMD), and a glucosyltransferase domain [8,9]. After receptor-mediated internalization and intracellular cleavage, the toxins glucosylate members of the Rho-Rac family of small GTPases at a specific threonine residue in host intestinal epithelial cells, leading to alterations in the actin cytoskeleton, massive fluid secretion, acute inflammation, and necrosis of the colonic mucosa [7]. Purified TcdA possesses potent enterotoxic and pro-inflammatory activity, as determined in ligated intestinal loop studies in animals [10,11]. TcdA is also cytotoxic to cultured cells in a picomolar to nanomolar range. TcdB, more cytotoxic to cultured cells than TcdA, was previously reported to exhibit no enterotoxic activity in animals [11,12], but recent studies have found enterotoxic and proinflammatory activities in human intestinal xenografts in severe combined immunodeficient (SCID) mice [13]. Furthermore, the TcdA^-^B^+ ^*C. difficile *strains are responsible for pseudomembranous colitis in some patients [14,15].

It is desirable to obtain relatively pure and biologically active TcdA and TcdB for studying the pathogenesis of CDAD and host immune response to the infection and for generating immunological tools for research and clinical diagnosis. The native toxins are usually purified from toxigenic *C. difficile *culture supernatant, which involves multiple steps and the purity is often unsatisfactory [16-18]. Attempts have been made to clone and express *C. difficile *toxins in *Escherichia coli *[19-21], but it is unclear whether or not purified toxins were obtained from the bacterial lysate in these studies. The Gram-positive *Bacillus megaterium *expression system may offer an alternative for the expression of *C. difficile *toxins due to several advantages over the *E. coli *system, including the lack of alkaline proteases activity and endotoxin LPS, capable of secreting expressed heterologous protein into the medium [22,23]. Although the expression level was low, Burger et al [24] were the first to successfully express and obtain the purified recombinant TcdA in *B. megaterium*. In this study, we have expressed the full-length of both TcdA and TcdB in *B. megaterium*. We were able to obtain an average of 5 – 10 mg of highly purified recombinant proteins from one liter of total bacterial culture. Both recombinant TcdA and TcdB were biologically active similar to their native purified toxins.

## Results

### Cloning and expression of the recombinant toxins

**Figure 1:**
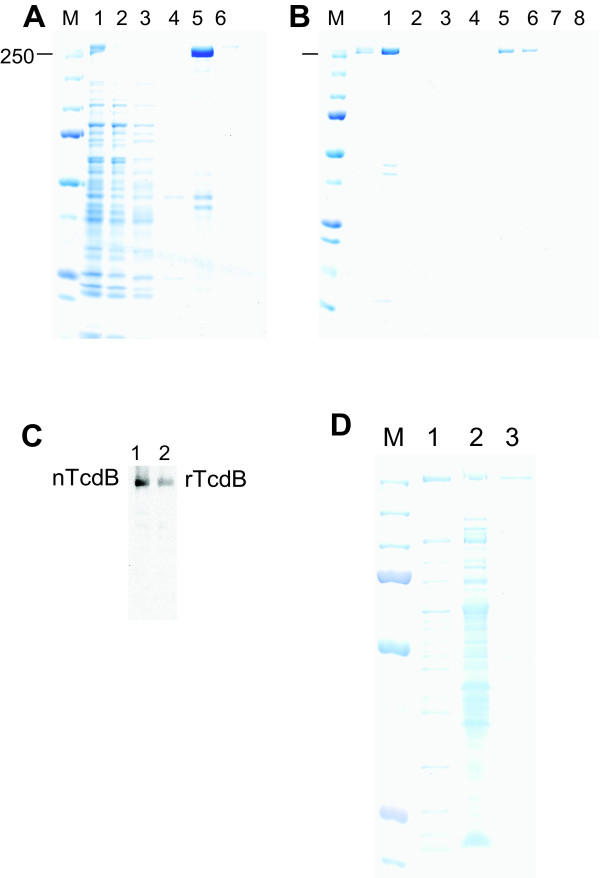
**Expression and purification of recombinant TcdB**. pHis-TcdB plasmid was transformed to *B. megaterium *protoplasts. Several transformed colonies were picked up and the expression of rTcdB was induced by xylose. (A) His-tag affinity purification of total lysate from *B. megaterium*. Lane 1: total bacterial lysate; Lane2: flow-through; Lane 3: wash; lane 4–6: elution fraction 1–3. (B) Anion-exchanging column fractionation after Ni-affinity chromatography. nTcdB: purified native TcdB from *C. difficile *culture supernatant. Lane 1: Elution fraction 2 from (A); Lane 2–8: fractions from a gradient salt elution. Fraction 5 and 6 contain purified rTcdB. (C) Western blot results of purified native TcdB and rTcdB of combined fraction 5 and 6 from (B). (D) Coomassie staining of SDS-PAGE from total sonication lysates a pHis-TcdB transformed clone (lane 1), concentrated supernatant from pHis-SP-TcdB transformed clone (lane 2) and purified native TcdB (lane 3). M indicates molecular weight marker and 250 kDa is showed.

After enzyme (BsrGI/KpnI) digestion of pHis1522 vector and PCR products, the ligation was set up and the mixture was incubated at 4°C for 2 days. The transformation was carried out following the standard protocol and more than 90 bacterial colonies were picked up. The plasmids (named pHis-TcdB) from these colonies were subjected to screening by digestion with a variety of enzymes. pHis-TcdB from multiple clones was used to transform *B. megaterium *protoplasts, and tetracycline-resistant clones were picked up. After induction with xylose, the bacterial crude extracts from approximately 30 different transformed clones were subjected to screening for activity causing rounding of cultured cells. The clones with high activities were selected and a Coomassie-staining gel showed expression of a protein of about 270 kDa when xylose was added for induction (Figure 1A Lane1). This protein was purified by Ni-affinity column from a total crude extract, resulting in a major band with molecular weight about 270 kDa and some weak contaminant bands of lower molecular weights (Figure 1A). A further step of ion-exchange purification resulted in a highly pure 270 kDa-protein without any visible contaminant bands on Coomassie-stained gels (Figure 1B). Western blot analysis using specific antibodies against His-tags (data not shown) and toxin B (Figure 1C) identified the 270 kDa protein from *B. megaterium *lysate as the recombinant His-tagged toxin B (rTcdB). From one liter of bacterial culture, approximately 10 mg of rTcdB was obtained. The pHis-TcdB used to transform the *B. megaterium *was subjected to DNA sequencing for the verification of *tcdB *gene, and no mutation was found (data not shown).

*B. megaterium *has a secretory pathway [22,23], which transports the expressed heterologous proteins into the medium. Because the native toxins are secreted into medium by toxigenic *C. difficile*, we attempted the expression of rTcdB in a secretory form. The DNA sequence encoding a 28-amino-acid signal peptide derived from *B. megaterium *extracellular esterase LipA [22] was synthesized and inserted at the 5' of tcdB. The resulting pHis-SP-TcdB was transformed into *B. megaterium*. Figure 1D shows the presence of a 270 kDa band from the concentrated bacterial culture supernatant on a Coomassie stained SDS-PAGE gel (lane 2). Western blot and cytotoxicity assays confirmed the expression of the secretory form of rTcdB. The expression level of this secretory form was, however, very low and we were unable to obtain a sufficient amount of purified rTcdB from the supernatant.

**Figure 2:**
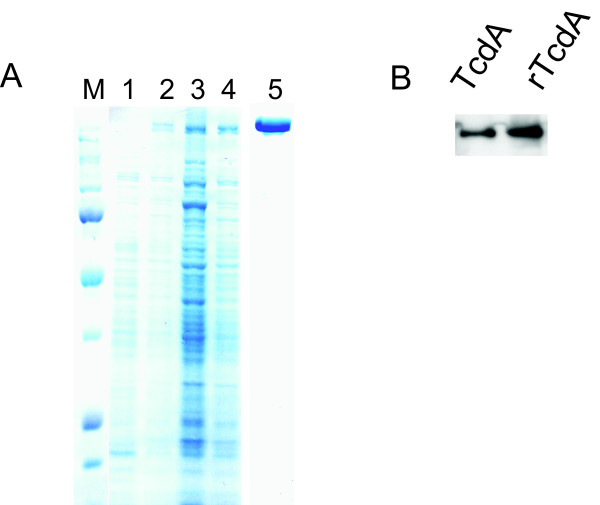
**Expression and purification of recombinant TcdA**. pHis-TcdA plasmid was transformed into *B. megaterium *protoplasts. Over 20 transformed colonies were picked up and the expression of rTcdA was induced by xylose. (A) Coomassie staining of SDS-PAGE from crude bacterial extracts: Lane 1 shows no xylose induction; Lane 2 to 4 shows three different clones with xylose induction; Lane 5 shows purified rTcdA after Ni-affinity and thyroglobulin chromatograph. M shows molecular weight marker and the top band indicates 250 kDa. (B) Western blot results of the purified native TcdA (lane 1) and rTcdA (lane 2).

A similar strategy was utilized to clone and express recombinant TcdA. On a Coomassie stained SDS-PAGE gel of total bacterial lysate, a clear protein band of 308 kDa appeared only upon induction with xylose in pHis-TcdA-transformed *B. megaterium *(Figure 2A). His-tag combined with thyroglobulin affinity purifications resulted in a single strong band (Figure 2A Lane 5), which was confirmed to be TcdA by western blot analysis (Figure 2B). The pHis-TcdA used to transform the *B. megaterium *was subjected to DNA sequencing for the verification of *tcdA *gene, and no mutation was found (data not shown).

### Cytopathic effect of recombinant toxins

**Figure 3:**
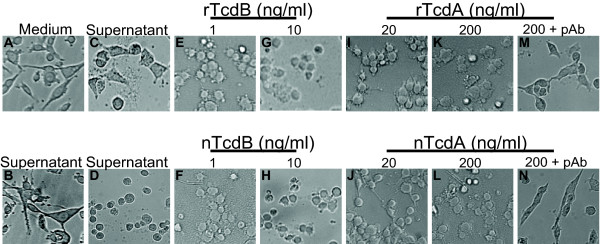
**Cytopathic effect of recombinant toxins**. Mouse colonic epithelial CT26 cells in a 96-well plate were untreated (A) or treated as following: 10 μl culture supernatant from pHis-TcdB-transformed *B. megaterium *without (B) or with (C) xylose induction; (D) 10 μl culture supernatant from pHis-SP-TcdB-transformed *B. megaterium *with xylose induction; (E) rTcdB (1 ng/ml); (F) native TcdB (1 ng/ml); (G) rTcdB (10 ng/ml); (H) native TcdB (10 ng/ml); (I) rTcdA (20 ng/ml); (J) native TcdA (20 ng/ml); (K) rTcdA (200 ng/ml); (L) native TcdA (200 ng/ml); (M) rTcdA (200 ng/ml) plus 1 μl/well of rabbit anti-TcdA serum; or (N) native TcdA (200 ng/ml) plus 1 μl/well of rabbit anti-TcdA serum. Cells were incubated overnight and the morphological changes were observed under a phase-contrast microscope.

We compared the cytopathic effects of recombinant and native toxins on cultured human epithelial HT-29 (data not shown) and mouse intestinal epithelial CT26 cells (Figure 3). rTcdA (Figure 3I and 3K) at 20 or 200 ng/ml caused CT26 cell rounding similarly to native TcdA (Figure 3J and 3L). Both rTcdB and native TcdB were more potent, capable of causing cell rounding at a much lower concentration than those of rTcdA or native TcdA (Figure 3E, F, G, and 3H). After xylose induction, both supernatant (Figure 3D) and total cell lysate (data not shown) from pHis-SP-TcdB transformed *B. megaterium *caused cell rounding, whereas the bacterial culture supernatants from pHis-TcdB transformed *B. megaterium *with (Figure 3C) or without (Figure 3B) xylose induction did not cause cell rounding. The same concentration of xylose in BHI medium did not cause any cell rounding (data not shown), suggesting the effects were specifically caused by the secreted toxin. Furthermore, the cytopathic effects of both rTcdA and native TcdA were blocked completely by a rabbit polyclonal antibody against TcdA (Figure 3M and 3N).

### Cytotoxic effect of recombinant toxins

**Figure 4:**
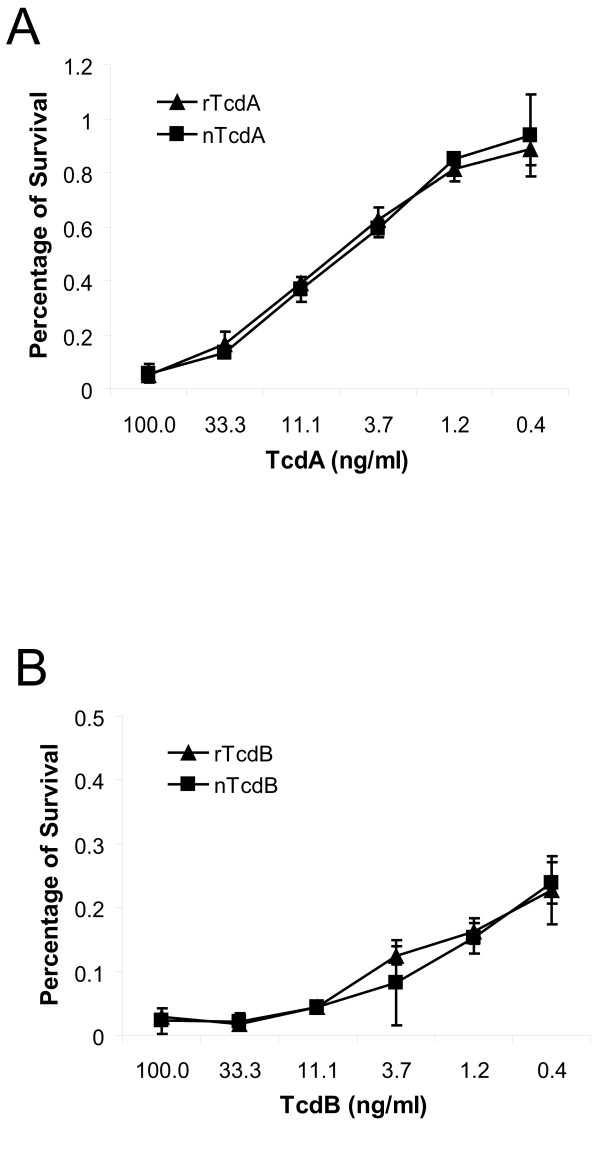
**Cytotoxic effect of recombinant toxins**. Mouse colonic epithelial CT26 cells in a 96-well plate were exposed to native (□) or recombinant (Δ) TcdA (A) or exposed to native (□) or recombinant (Δ) TcdB (B) for 72 hr. The MTT assay was performed and cell viability was expressed as the percentage of survival cells without exposure to toxins.

It has been demonstrated that both TcdA and TcdB induce intestinal epithelial cells to undergo apoptosis [25-27]. We next compared the cytotoxicity of recombinant toxins to their native counterparts on cultured epithelial cells. As shown in Figure 4A, both native and recombinant TcdA induced a comparable and dose-dependent cell death of CT26 cells. Similarly, the exposure of CT26 cells to rTcdB induced a comparable cell death with nTcdB as determined by MTT assay (Figure 4B). These data indicate that the recombinant TcdA and TcdB have similar cytotoxic activity to their native counterparts in CT26 cells.

### Glucosylation of Rac1 by recombinant toxins

**Figure 5:**
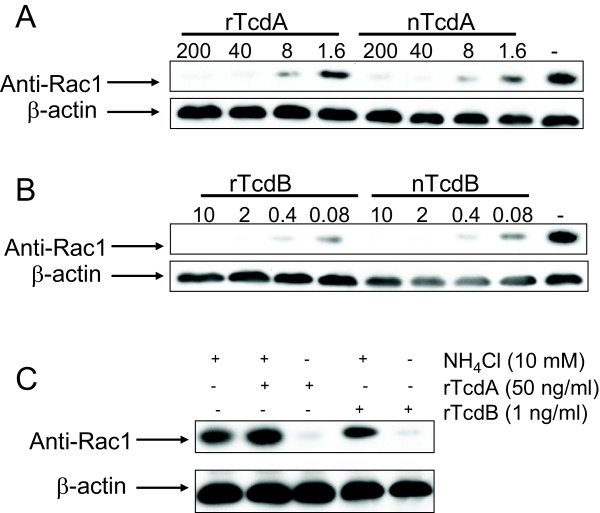
**Glucosylation of Rac1 by recombinant toxins**. CT26 cells were either untreated or treated with the indicated amount of native or recombinant TcdA (A) and TcdB (B) for 5 hr. (C) CT26 cells were pretreated with ammonia chloride for 30 min before the exposure of the indicated amount of recombinant toxins for 5 hr. Cells were harvested and western blot was performed as described in the Materials and Methods. Monoclonal antibody clone 102 recognizes unglucosylated Rac1 and has a reduced affinity to glucosylated Rac1.

*C. difficile *TcdA and TcdB target host cell Rho GTPase by glucosylating these proteins at the specific Thr37 (Rho A) or Thr35 (CDC42 and Rac1) residues [28,29]. To determine whether recombinant toxins can glucosylate Rac1 protein of host cells, mouse intestinal epithelial CT26 cells were treated with recombinant or native TcdA and TcdB for 5 hr. Western blot showed that both rTcdA and nTcdA treatment induced a dose-dependent, reduced recognition of Rac1 by monoclonal antibody (anti-Rac1 clone 102) that recognizes non-glucosylated Rac1 [30] (Figure 5A). The reduced recognition of Rac1 was not the results of protein degradation, because total Rac1 protein remained unchanged as determined by western blot with an antibody (clone 23A8) recognizing both glucosylated and unmodified Rac1 (data not shown). Both rTcdA and nTcdA at 40 ng/ml resulted in a complete glucosylation of Rac1 whereas 8 or 1.6 ng/ml of TcdA led to a partial glucosylation. rTcdB or nTcdB exposure resulted in a completely glucosylation of Rac1 at a dose range from 0.4 – 2 ng/ml (Figure 5B), indicating that the glucosyltransferase activity of TcdB was more potent than TcdA. These results were consistent with the activity of native *C. difficile *toxins in which TcdB is more toxic to cultured cells than TcdA. The treatment of rTcdA and rTcdB also led to the glucosylation of Rac1 in RAW 264.7 and CHO cells (data not shown).

Both TcdA and TcdB are thought to bind to specific cellular receptor(s), which mediate their cellular uptake through endocytosis [31-33]. Lysosomotropic agents such as chloroquine and NH_4_Cl can inhibit toxin-mediated cytotoxicity, suggesting that the endosomal acidification is involved in toxin intracellular trafficking [34,35]. To determine whether or not the cellular activity of the recombinant toxins also require endosomal acidification, CT26 cells were pre-incubated with NH_4_Cl for 30 min before the toxin exposure. While the exposure of CT26 cells to rTcdA or rTcdB induced the glucosylation of Rac1, pretreatment of cells with ammonium chloride completely blocked such an activity (Figure 5C).

### Disruption of tight junctions of intestinal epithelial cells by recombinant toxins

**Figure 6:**
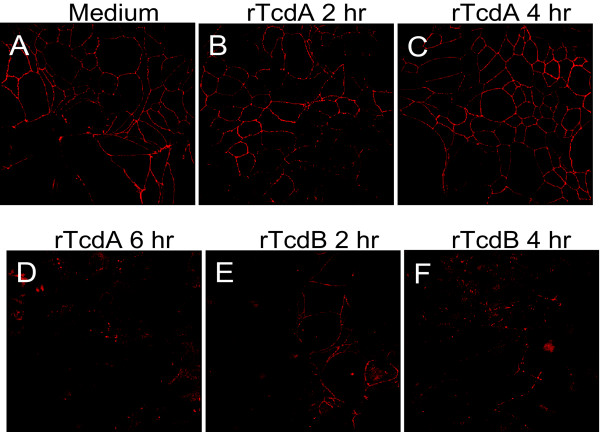
**Disruption of tight junctions by recombinant toxins**. HCT-8 cells on transwells were cultured untill the formation of tight junctions. The polarized monolayers were untreated (A) or treated with 300 ng/ml of rTcdA for 2 hr (B), 4 hr (C), and 6 hr (D); or with rTcdB 300 ng/ml for 2 hr (E), and 4 hr (F). Cells on the transwell membrane were fixed and stained with anti-oocludin and fluorochrome-conjugated secondary antibodies, and then visualized under a confocal microscope.

It is known that TcdA and TcdB alter the structure of intestinal epithelia by disrupting tight junctions [36]. We investigated whether or not the recombinant toxins we generated have similar activities. Human intestinal epithelial HCT-8 cells were cultured in a transwell for 10 to 14 days until transepithelial resistance (TER) reached 1000 Ω*cm^2 ^and tight junctions were formed (Figure 6A). rTcdA did not alter cell-cell connections of HCT-8 cells within 4 hr of treatment (Figure 6B and 6C) but disrupted tight junctions after 6 hr of treatment (Figure 6D). rTcdB, at the same concentration with rTcdA, completely disrupted the tight junctions of HCT-8 within 2 hr of treatment (Figure 6E and 6F).

## Discussion

CDAD is largely attributed to the bacterial exotoxins TcdA and TcdB, although other virulence factors probably also play some roles [7]. Both toxins glucosylate Rho GTPase family proteins, leading to their inactivation [1,7]. To study the pathogenesis of CDAD and host immune response to the toxins, and to use the toxins as a research tool for signal transduction involving Rho GTPases, it is necessary to obtain large quantities of highly purified active TcdA and TcdB. Native toxins can be purified from toxigenic *C. difficile *culture supernatant. The purification process however usually involves multiple steps such as ultrafiltration, ammonium sulfate precipitation, and ion-exchange chromatography [17,18]. In the case of TcdA, affinity purification through thyroglobulin is available and relatively pure TcdA can be obtained ([16] and unpublished data). TcdB however is often contaminated with some unknown substance even after extensive purification steps ([18] and unpublished data). Gene engineering to generate recombinant toxins seems to be a logical approach for obtaining large quantities of pure toxin proteins. The *E. coli *system is popular and is widely used for the expression of recombinant proteins. Several studies have reported to have cloned and expressed active *C. difficile *toxins in *E. coli*, but failed to demonstrate that the purified toxins were obtained from bacterial extracts [19-21]. Burger *et al *was the first to report a successful expression of recombinant TcdA using *Bacillus megaterium *system [24]. Recombinant TcdB, however, has not yet been stably expressed. In this study, we were successfully expressing a full-length recombinant TcdB in *B. megaterium *and greatly enhanced the expression level of recombinant TcdA.

Because *E. coli *expression system is widely used and easy-to-handle, we have utilized various *E. coli *expression vector systems to clone and express both toxins and found that both recombinant TcdA and TcdB were expressed in *E. coli *but we were unable to obtain the purified toxins from the bacterial lysate (unpublished data). The large size of the heterologous proteins may possibly be prone to protease digestion, and intrinsic protease activities of these toxins [37,38] may also contribute to the instability. Another possible reason for the inability to obtain purified recombinant toxins from *E. coli *is that the expression level was too low. In fact, large proteins are difficult to express at high levels in *E. coli*. Furthermore, an unusually high content of AT (~70%) in these clostridial gene sequences and a different codon -usage make them particularly difficult to express at a high level in *E. coli *[39,40].

There are several advantages associated with *B. megaterium *expression system including lack of alkaline phosphatase and endotoxin LPS. More importantly, *B. megaterium *has been successfully used to express rTcdA [24], we therefore decided to use this system. Initially, we followed the reported cloning strategy but we were unsuccessful. This might be due to the extremely low ligation efficiency [24] when putting several fragments together during the cloning. We then changed our strategy to a direct PCR amplification of whole toxin genes. However, this strategy was associated with a potential risk that PCR might introduce mutations due to the large size of the genes (7101 bp and 8133 bp for *tcdB *and *tcdA *respectively). Because of the large size of both genes, it was impossible to sequence all constructs in order to identify the correct clones. We therefore took a different approach, screening for recombinant protein expression and cytotoxicity. We screened as many colonies as possible (more than 90) after ligation (data not shown) and then transformed these constructed shuttle vectors into *B. megaterium *protoplasts. The resulting colonies were subjected to screening again by examining the cytotoxic activity in bacterial crude extracts. The specific expression of recombinant toxins from the positive colonies (with cytotoxic activity) was then determined by western blot. The plasmids encoding *tcdA *or *tcdB *respectively were finally subjected to DNA sequencing and no single mutation was found in either of the toxin genes.

The average yields of recombinant TcdA and TcdB were about 5 – 10 mg/liter of bacterial culture, which were much higher than that of native toxins from *C. difficile *culture, and up to 20-fold higher than the amount of rTcdA reported by Burger et al [24]. The fact that we used a different shuttle vector from that of Burger et al [24] might explain our higher yields of recombinant proteins. In addition, we have found out a twelve- to sixteen-hour period of induction with xylose yielded more recombinant proteins than five hours as suggested by the manufacturer. The other possible explanation was that the *tcdA *gene was fused into *orf1 *of the vector in the construct reported by Burger et al [24], resulting a modified N-terminus of recombinant TcdA. This modification might affect the efficiency of protein expression and/or its stability. We inserted the *tcdA *or *tcdB *gene into the BsrGI site and the protein translation directly started from toxin gene, resulting in recombinant toxins without any N-terminal modification.

To facilitate purification, a His_6 _tag was fused to the C-terminus of both toxins. One-step of Ni-affinity chromatography resulted in approximately 70–80% purity of recombinant toxins as determined by Coomassie staining of SDS-PAGE (Figure 1 and data not shown). To achieve a greater purity, an additional step was introduced after Ni-affinity chromatography. For rTcdA, thyroglobulin affinity purification was used. rTcdB was further purified by an anion-exchange fractionation (Figure 1B). These additional purification steps for rTcdA and rTcdB, respectively, led to highly pure recombinant toxins. No additional band was seen on a Coomassie-stained gel (Figure 1B for rTcdB, and Figure 2A for rTcdA). In addition, there were no detectable TLR2 or TLR4 stimulants within the purified recombinant toxins as determined by sensitive bioassays (unpublished data). This is important because any use of the impure proteins to study host immune response or signaling transduction would possibly result in unintended consequences.

Unlike *E. coli*, a protein secretory pathway is present in *B. megaterium *[22,23]. A 28-amino-acid signal peptide of *B. megaterium *extracellular esterase LipA can direct the transport of expressed heterologous proteins into medium and the sequence is removed by a protease after secretion [22]. Because of the nature of exotoxins of native toxins and relatively easy purification from supernatant without the need for sonication or other means of cell disruptions, we sought to express rTcdB extracellularly. After inserting the gene of the leader signaling peptide at the 5' of *tcdB*, we were able to generate active secretory rTcdB (Figure 3D) and Coomassie staining of concentrated supernatant on a SDS-PAGE gel and western blot showed rTcdB band (Figure 1D and data not shown). However, for some unknown reasons, the expression level of rTcdB was significantly decreased when the leader sequence was introduced to the construct. In addition, we found the secretion was very inefficient and the majority of toxins were still trapped inside of the bacteria, since the total bacterial lysate contained the majority of toxin activity (data not shown). This secretory pathway may not be efficient for such a large size of protein. The low amount of rTcdB in the medium might also be due to the inefficient refolding after their extracellular transport.

## Conclusion

In this study, we have successfully expressed the full-length of recombinant *C. difficile *toxin A and B proteins in *B. megaterium *and greatly improved the yield of the recombinant toxins. We were able to obtain 5 – 10 mg of highly purified rTcdA or rTcdB from one liter of bacterial culture. These recombinant toxins had similar biological activities with their native counterparts. We believe the recombinant toxin proteins will provide an invaluable tool for studying the pathogenesis of *C. difficile *associated disease and host immune response to toxins, as well as tools for studying the signaling transduction involving Rho GTPase family proteins.

## Methods

### Cell lines

Human intestinal epithelial cell lines HCT-8 and mouse colonic epithelial cell CT26 were purchased from ATCC (Manassas, VA). All these cells were cultured in DMEM medium (Invitrogen, Carlsbad, CA) containing 2 mM L-glutamine, 100 U/ml penicillin G, 50 μg/ml streptomycin sulfate, and 10% fetal bovine serum.

### Purification of native toxins

Native TcdA and TcdB were purified from *C. difficile *strain VPI 10463 (kindly provided by Abraham L. Sonenshein, Tufts University School of Medicine) as described previously [41]. Briefly, a dialysis bag (100 kDa cutoff, Millpore, Billerica, MA) containing 100 ml of 0.9% NaCl immersing in a total volume of 1 liter of brain heart infusion (Difco, Lawrence, KS) was inoculated with 5 ml of an overnight culture of *C. difficile*, and the culture was grown in an anaerobic chamber (Bactron BACLITE-1, Sheldon Manufacturing Inc., Cornelius, OR) at 37°C for 72 hr. The supernatant collected from the dialysis bag was concentrated by ultrafiltration through an Amicon XM-100 membrane (Millipore). The concentrated supernatants were dialyzed against Tris-HCl (50 mM, pH 7.5) buffer overnight and loaded onto a HiTrap DEAE column (Amersham Biosciences, Piscataway, NJ). The fractions containing toxins were collected and then passed through a thyroglobulin column, and the flow-through was further passed through a Mono Q column (Amersham Biosciences). The elutions from thyroglobulin and mono Q yielded TcdA and TcdB respectively.

### Constructs and cloning

The tcdB gene was amplified from *C. difficile *(VPI 10463) chromosomal DNA using forward primer 5'-GCGCTGTACAATGAGTTTAGTTAATAGAAAAC-3' and reverse primer 5'-ATATATGGTACCCTTCACTAATCACTAATTGAGC-3'. The PCR product was digested by BsrGI and KpnI enzymes, and then ligated to pHis1522 vector (MoBiTec, Goettingen, Germany). The full-length of *tcdA *gene was amplified using the primers 5'-GCGCTGTACAATGTCTTTAATATCTAAAGAAGAGTTAA-3' and 5'-ATATGCATGCCCATA TAT CCCAGGGGCTTTTA-3'. The PCR product was digested by BsrGI and SphI, and then inserted into pHis1522 vector. Both sequences of *tcdA *and *tcdB *genes in pHis 1522 vector have been confirmed by DNA sequence using a panel of primers (Table 1). The gene encoding a 28-amino-acid signal peptide of *B. megaterium *extracellular esterase LipA [22] that directs protein secretion in the secretory pathway of *B. megaterium *was synthesized by GeneArt (Regensburg, Germany) and inserted at the site of BsrGI of the *tcdB *construct. All restriction endonucleases were purchased from New England Biolabs (Cambridge, MA). All DNA cloning and plasmid construction were performed at Tufts University and approved by the Institutional Biosafety Committees and conformed with NIH Recombinant DNA technology guidelines.

**Table 1:** The sequences of DNA sequencing primers

		*tcdA *Sequencing Primers
		**Sequence**
TcdA-Seq1	Forward	CTGCAGCATCTGACATAG
TcdA-Seq2	Forward	AAGTTATGAAGCAACATGC
TcdA-Seq3	Forward	TCATCTCCATCTATAAGTTCTC
TcdA-Seq4	Forward	GTTTCTGGAAATTGTTTGG
TcdA-Seq5	Forward	GTTACTGGATGGCAAACC
TcdA-Seq6	Reverse	TAGTCCAATAGAGCTAGGTC
TcdA-Seq7	Reverse	CCATGTCCAATAAAGGTTAC
TcdA-Seq8	Reverse	ACTGCTCCAGTTTCCCAC
TcdA-Seq9	Reverse	ACATTTCTACCATTTCCG
TcdA-Seq10	Reverse	ATAACCAGTTGAGGCTATG

		*tcdB *Sequencing Primers
		**Sequence**

TcdB-Seq1	Forward	GAACAAGAGTTGGTAGAAAG
TcdB-Seq2	Forward	TCTTGGTGAAGATGATAATC
TcdB-Seq3	Reverse	CCTGGTAACATATCAACATC
TcdB-Seq4	Reverse	CTCTCTCTGAACTTCTTGC
TcdB-Seq5	Forward	CCTACATTATCTGAAGGATTAC
TcdB-Seq6	Forward	GATGTTGATAATGTTGTGAGAG
TcdB-Seq7	Forward	ATAGTAAGCCTTCATTTGG
TcdB-Seq8	Reverse	GCTGCACCTAAACTTACAC
TcdB-Seq9	Reverse	ATTACTTCCATTTACCTCAC
TcdB-Seq10	Forward	TTATAGAGGAGCTGTAGAATG
TcdB-Seq11	Reverse	GCTTTACCTGTTTCTGGG

		Sequencing Primers in pHis-1522 vector

		**Sequence**
phis-seq-F	Forward	TTTGTTTATCCACCGAACTAAG
phis-seq-R	Reverse	TGATTGGCTCCAATTCTTG

### Expression of recombinant toxins

The transformation of *Bacillus megaterium *protoplasts were performed according to the manufacturer's instruction (MoBiTec). The transformed *B. megaterium *colonies were picked up and grown overnight in an LB medium supplemented with 10 μg/ml tetracycline. The overnight cultures were diluted 1:30 in LB medium containing tetracycline and grown to an optical density (OD_600_) around 0.3 before the addition of xylose (0.5% w/v) for inducing protein expression. Bacteria were harvested by centrifugation after 12 to 16 hr of induction. In case of the secretory rTcdB, the culture supernatant was also collected.

### Purification of recombinant toxins

Purification of recombinant His-tagged rTcdB from bacterial lysate was performed by Ni-affinity chromatography following an ion-exchange fractionation. Briefly, the *B. megaterium *culture pellet (from 100 ml of culture) was resuspended in 5 ml lysis buffer (300 mM NaCl, 20 mM imidazole, 20 mM NaH_2_PO_4_, 500 μM EDTA, protease inhibitor cocktail (Cat #P8849, Sigma), adjusted to pH 8.0). Cells were disrupted by sonication and the lysate was centrifuged at 14,000 *g *for 20 min. The supernatant was then passed through a nickel-charged HiTrap chelating HP column (Amersham Biosciences, Piscataway, NJ) and the bound His-tagged rTcdB was eluted with an elution buffer containing 250 mM imidazole, 300 mM NaCl, and 20 mM NaH_2_PO_4_, pH 8.0. The eluent was then desalted and applied to HiTrap Q column (Amersham Biosciences) and rTcdB was eluted by a gradient concentration of NaCl solution. The fraction containing rTcdB was combined and stored at -80°C until use. To purify rTcdB from supernatant, the bacterial supernatant was passed through a 0.45 mm filter first, and then concentrated through ultrafiltration with a 100 kDa cutoff membrane (Millipore). The concentrated supernatant was then performed with Ni-affinity chromatography following an ion-exchange fractionation as described above. rTcdA was purified by Ni-affinity chromatography following an thyroglobulin affinity column. The method for thyroglobulin purification has been described elsewhere [16].

### SDS-PAGE and western blot

CT26 cells were exposed to toxins for 5 hr before they were harvested. In other experiments, ammonium chloride was pre-incubated with the cells for 30 min before the cells were exposed to toxins. The methods for SDS-PAGE and western blot have been described previously [42,43]. Briefly, the samples were boiled for 5 min in 1× NuPage SDS sample buffer (Invitrogen) and then loaded on a gradient (4 – 20%) sodium dodecyl sulfate-polyacrylamide gel (SDS-PAGE) (BioRad, Hercules, CA). After electrophoresis, the gels were stained with GelCode Blue (Pierce, Rockford, IL) according to the manufacturer's instructions. For western blot, TcdB-specific monoclonal antibody (clone 5A8-E11, Meridian Life Science, Inc., Memphis, TN), and TcdA-specific monoclonal antibodies (clone PCG4.1, Meridian Life Science, Inc.; or clone A1E6 generated in our laboratory) and HRP-conjugated anti-mouse IgG (Amersham Biochiences) were used as the primary and secondary antibodies respectively and the protein bands were visualized by an enhanced chemiluminescence assay (ECL, Amersham Biochiences). To determine Rac1 glucosylation, the antibody (clone 102, BD Biosciences, San Diego, CA) specific against non-glucosylated form of Rac1 was used. Anti-actin antibody (clone AC-40, Sigma, St. Louis, MO) was used to monitor an equal loading of samples.

### Cytotoxicity assay

Subconfluent CT26 cells (2 ×10^4^/well) seeded in 96-well plates were incubated with toxins, bacterial culture supernatants, or crude extracts. For blocking experiments, 1 μl/well of rabbit anti-serum against TcdA was added simultaneously with toxins. Cells were cultured overnight and the morphological changes of CT26 cells were observed by light microscopy with a CCD camera. For the MTT assay, after 3 days of incubation, 10 μL of MTT (5 mg/ml) were added to each well and the plate was further incubated at 37°C for 2 h. The formazan was solubilized with acidic isopropanol (0.4 N HCl in absolute isopropanol), and absorbance at 570 nm was measured using a 96-well ELISA reader. Cell viability was expressed as the percentage of survival of the control wells.

### Disruption of tight junctions by recombinant toxins

HCT-8 cells were seeded into a 24-well plate with 3-μm pore transwells (Corning Inc., Wilkes Barre, PA) and cultured for 10 to 14 days. Transepithelial resistance (TER) was monitored daily until TER reached 1000 Ω/cm^2^. rTcdA (300 ng/ml) or rTcdB (300 ng/ml) was added into the upper chamber of transwells for the indicated time. The cells were fixed and stained with anti-occludin (clone OC-3F10, Invitrogen) and fluorochrome-conjugate secondary antibodies. The slides were examined under confocal microscope (Leica LSM TSC SP2 AOBS).

## Authors' contributions

GY and BZ carried out the molecular cloning, design of constructs, DNA sequencing, and data preparation. JW carried out protein purification. XH established the methods for protein purification. XS and WN participated in molecular cloning. ST contributed intellectually to experimental design and text. HP initiated the project, conceived the whole study and experimental design, and drafted the manuscript. All authors read and approved the final manuscript.
